# Carboxymethylcellulose reinforced starch films and rapid detection of spoiled beverages

**DOI:** 10.3389/fbioe.2022.1099118

**Published:** 2023-01-06

**Authors:** Shijiao Qin, Hao Sun, Xiaoli Wan, Yujia Wu, Xu Lin, Huan Kan, Defa Hou, Zhifeng Zheng, Xiahong He, Can Liu

**Affiliations:** ^1^ National Joint Engineering Research Center for Highly-Efficient Utilization Technology of Forestry Resources, Southwest Forestry University, Kunming, China; ^2^ Lincang Academy of Forestry Sciences, Lincang, China; ^3^ College of Energy, Xiamen University, Xiamen, China

**Keywords:** sodium carboxymethyl cellulose, starch films, pH-responsive, rapid detection, food quality

## Abstract

The integrity of the packaging of a liquid foodstuff makes it difficult to detect spoilage. Therefore, it is important to develop a sensitive, fast and real-time material for liquid food detection. CMC, as lignocellulose derivatives and starch are widely used in the food industry. In this study, starch films with pH-responsive properties are successfully prepared from full-component starch and corn amylopectin (CA) by adding CMC. The effects of CMC on the mechanical properties, morphology characteristics, physical and chemical structures, stability and pH responsiveness of the starch films are analyzed. The starch/CMC-1.0 g composite films display good electrical conductivity and reduce the resistance of the composite film by two orders of magnitude. The composite films have pH response ability; in the simulation of orange juice spoilage experiment, the CA/CMC composite film has a more sensitive current response and was more suitable for the application to liquid food quality detection. Additionally, the starch/CMC composite films have potential applications for rapid detection and real-time monitoring of the safety of liquid food.

## 1 Introduction

With the rapid development of society and the economy, the issue of food safety is deeply rooted in people’s minds and is closely related to food safety, the social economy and human life (Serge et al., 2022; Jin and Zhong, 2022; He et al., 2017; Seto and Ramankutty, 2016; Liu and Hou, 2022). Food safety problems are mainly caused by the following factors: pesticide residues (Tang et al., 2021), bacterial species and biotoxins (Yesim et al., 2022), heavy metal contamination (Xiang et al., 2021), and illegal use of food additives (Wang et al., 2021). Existing food quality testing techniques mainly adopt spectroscopic methods, such as near-infrared spectroscopy (Wafula et al., 2022) and Raman spectroscopy (Simona et al., 2021). Although these techniques are highly accurate, the detection instruments are expensive, the detection time is long, and experienced personnel is required. However, these techniques cannot achieve the goal of rapid detection and real-time monitoring. The main techniques commonly used for rapid food detection are physicochemical, enzymatic (Martins et al., 2022), and immunoassay analyses (Xu et al., 2018) and bioluminescence (Ibarra et al., 2021). Although the above analytical techniques are applied for rapid detection, they have drawbacks, such as environmental impacts on the detection results and false positives. These techniques cannot simultaneously achieve rapid, simple, real-time and accurate food safety detection. Therefore, it is vital to develop rapid and sensitive food detection methods and functional materials.

Since juice is a liquid food, the integrity of the packaging makes it difficult to detect changes in its quality. Hence, it is important to develop a fast, real-time liquid detection material for this characteristic. The mechanical properties and stability of a film as a detection material are of great significance. Accordingly, researchers often develop film materials equipped with the desired functional properties by doping with other substances. Sodium carboxymethyl cellulose (CMC), a derivative of cellulose, has the characteristics of polyelectrolyte properties and good biocompatibility; the films prepared with CMC and starch have functional properties. The thermoplastic starch/CMC composite film prepared by Behera et al. (2022) showed that the mechanical properties and barrier properties of the original starch film were improved with the doping of CMC. Wi et al. (2011) demonstrated that intermolecular interactions between CMC and tapioca starch improve the tensile strength of the composite films, reduce their water solubility, and increase hydrophobicity using Fourier transform infrared (FTIR) spectroscopy. Aytunga et al. (2017) discovered that CMC/starch composite films have better transparency and water vapor barrier properties after adding glycerol. Rungsiri et al. (2019) prepared composite films from self-extracted CMC with rice starch and found that the doping of CMC not only improved the mechanical properties of the films but also enhanced the thermal stability. Lan et al. (2020) added Lactococcus lactis to CMC/starch composite films to give them antibacterial properties, and the optimal mechanical properties were obtained when the mass ratio of CMC to starch was 1:1. Jiang et al. (2020) reported an intelligent responsive packaging material for food quality monitoring. Based on the sensitivity of anthocyanins to pH and volatile ammonia from protein decomposition, the CMC/starch composite film with anthocyanins added can be used to assess the environmental pH and the freshness of fish. The above literature demonstrates that CMC enhances the mechanical properties and stability of starch films and provides a stable carrier for the pH responsiveness of the film.

In foodstuffs, the deterioration of liquid food mostly changes its pH. Therefore, the special pH signal allows the development of intelligent indicator materials suitable for real-time detection. Zhang et al. (2020) developed a label for detecting food spoilage based on the pH sensitivity of the indicator label. The label not only detects the pH of liquid food but also monitors in real time the level of biogenic amines produced by decaying food. In food detection technology, the combination of electrochemical parameters enables the visualization of food spoilage. Deng et al. (2013) proposed a graphene-modified acetylene black paste electrode for the detection of BPA concentration in food with a wide detection range and high sensitivity. Kundan et al. (2019) produced an ultra-sensitive optofluidic-surface enhanced Raman scattering (SERS) sensor for the detection of the food additive rhodamine 6G and the response to trace amounts of chlorobenzene compounds in drinking water. They revealed that smart electrical signal responsive materials have a wide range of applications and show extremely broad use in food quality detection. The growing demand for fruit and vegetable beverages has made the issue of food safety in this field crucial. Thus, the search for a simple, rapid, highly sensitive, real-time method for monitoring fruit and vegetable beverages is imperative.

**FIGURE 1:**
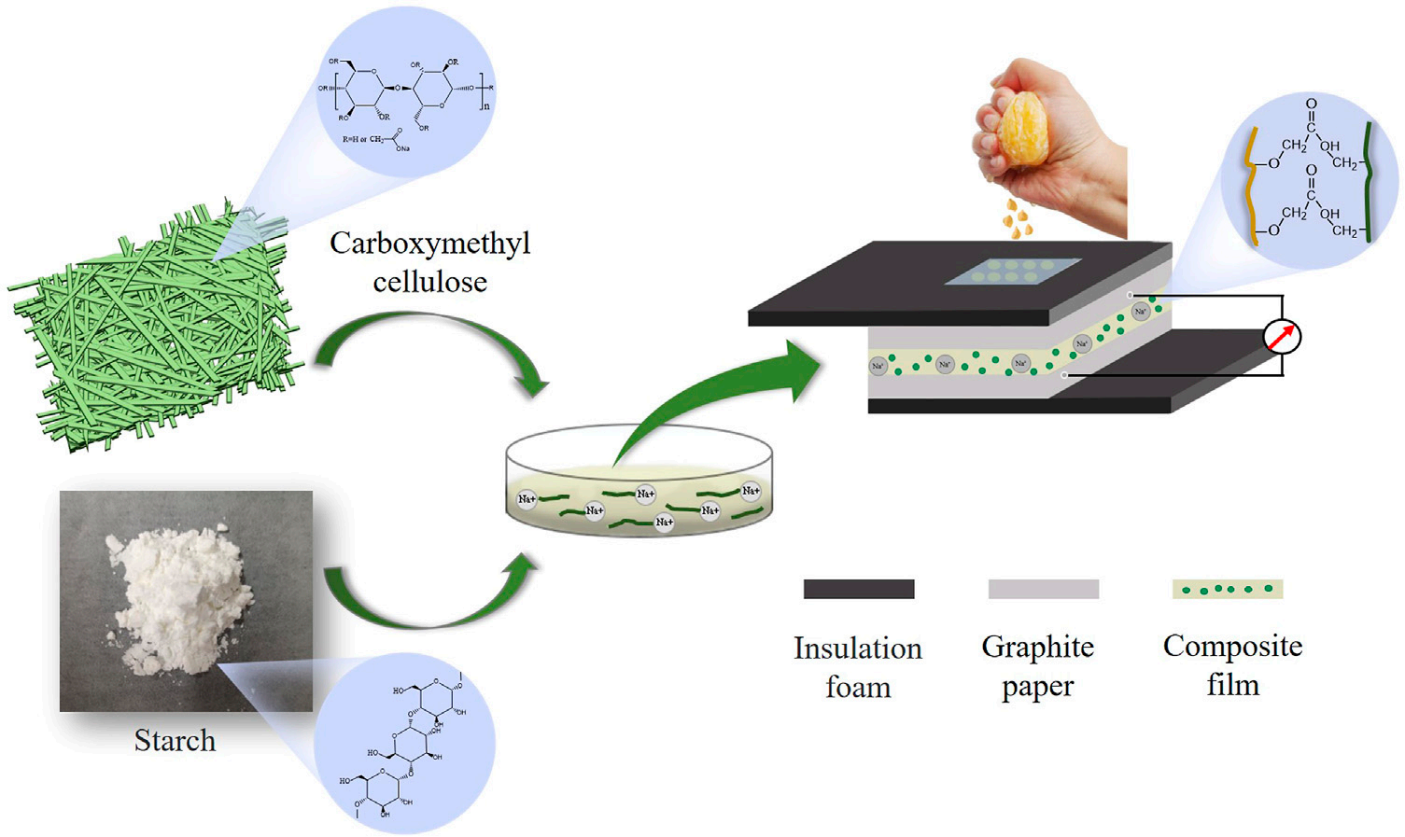
Schematic diagram of the structure of the starch/CMC composite film applied to the rapid detection of juice quality.

Considering the above issues, we prepared a composite functional film with starch as the matrix, glycerol as the plasticizer and CMC as the additive; these films provide rapid feedback of electrical signals to the pH of the juice (Figure 1). The mechanical properties, stability performance, and pH response characteristics of the composite film were examined. Based on the above analytical methods, we compared the effect of CMC on starch films with different amylopectin contents and discussed the influence trend of CMC addition on the functions of the composite films. It was found that the composite films responded sensitively to the pH of fruit juice. This study provides a new method for rapid, sensitive and non-destructive monitoring of the quality of liquid foodstuffs; this has highly promising applications in the field of rapid, non-destructive and real-time online monitoring of food quality.

## 2 Materials and methods

### 2.1 Experimental materials

Corn starch (CS), corn amylopectin (CA) and sodium carboxymethyl cellulose (CMC) were purchased from Shanghai Aladdin Biochemical Technology Co., AR grade. Ultrapure water was used throughout this study.

### 2.2 Preparation process

The starch/CMC composite films were prepared by solution casting. For preparation of 4% starch aqueous solution, 4 g starch and 1.4 g of glycerol (35% of the starch), which was used as a plasticizer, were weighed (mixing time 30 min). This was then transferred to a 90°C water bath and stirred until completely gelatinized (gelatinized for 1 h). Different masses (0, .2, .4, .6, .8, and 1.0 g) of CMC were added to the starch solution and stirred again. The homogeneous film solution (34 g) was poured onto Petri dishes (d = 15 cm) and baked in a constant temperature oven at 45°C for 6 h. The composite film was labeled as starch/CMC-x, where x indicated the amount of CMC added to the starch film. For example, CS/CMC-1.0 means that the amount of CMC doped in the starch film is 1.0 g.

### 2.3 Characterization and analysis

The component interaction of the sample was analyzed using FTIR (model: is 50 FT-IR). The attenuated total reflectance (ATR) method was used to scan from 4,000 to 600 cm^−1^ with a total of 32 scans per sample. X-ray photoelectron spectroscopy (XPS) was performed using a Thermo Scientific K-Alpha X-ray photoelectron spectrometer. X-ray diffraction (XRD) was performed using a Japanese Neo-D/MAX220 instrument with Cu as the target material; the relative crystallinity was analyzed using Jade six software. The morphology and microstructure of the films were examined by scanning electron microscopy (SEM). An intelligent electronic tensile testing machine (model: XLM (PC)) was used to measure the mechanical properties. Thermogravimetry-differential thermogravimetry (TG-DTG) was performed using a TGA209 F3. The samples (3–5 mg) were weighed, and the heating rate was 20 K/min under a N_2_-atmosphere. A contact angle tester (JC 2000D3R) was used to evaluate the surface hydrophilicity of the films; the impedance and chronoamperometry of the film samples were measured using a CHI760E electrochemical workstation. The test frequency was .1 Hz–1 MHz, and the amplitude was .01 V. Layer-by-layer self-assembled devices were produced for electrochemical testing. The device was prepared by coating graphite paper and the innermost film sample with an insulating collodion. The measure liquid was added dropwise to the surface of the film; the steady current and resistance were determined using chronoamperometry and electrochemical impedance spectroscopy (EIS).

## 3 Results and discussion

### 3.1 Mechanical properties of different composite films

**FIGURE 2:**
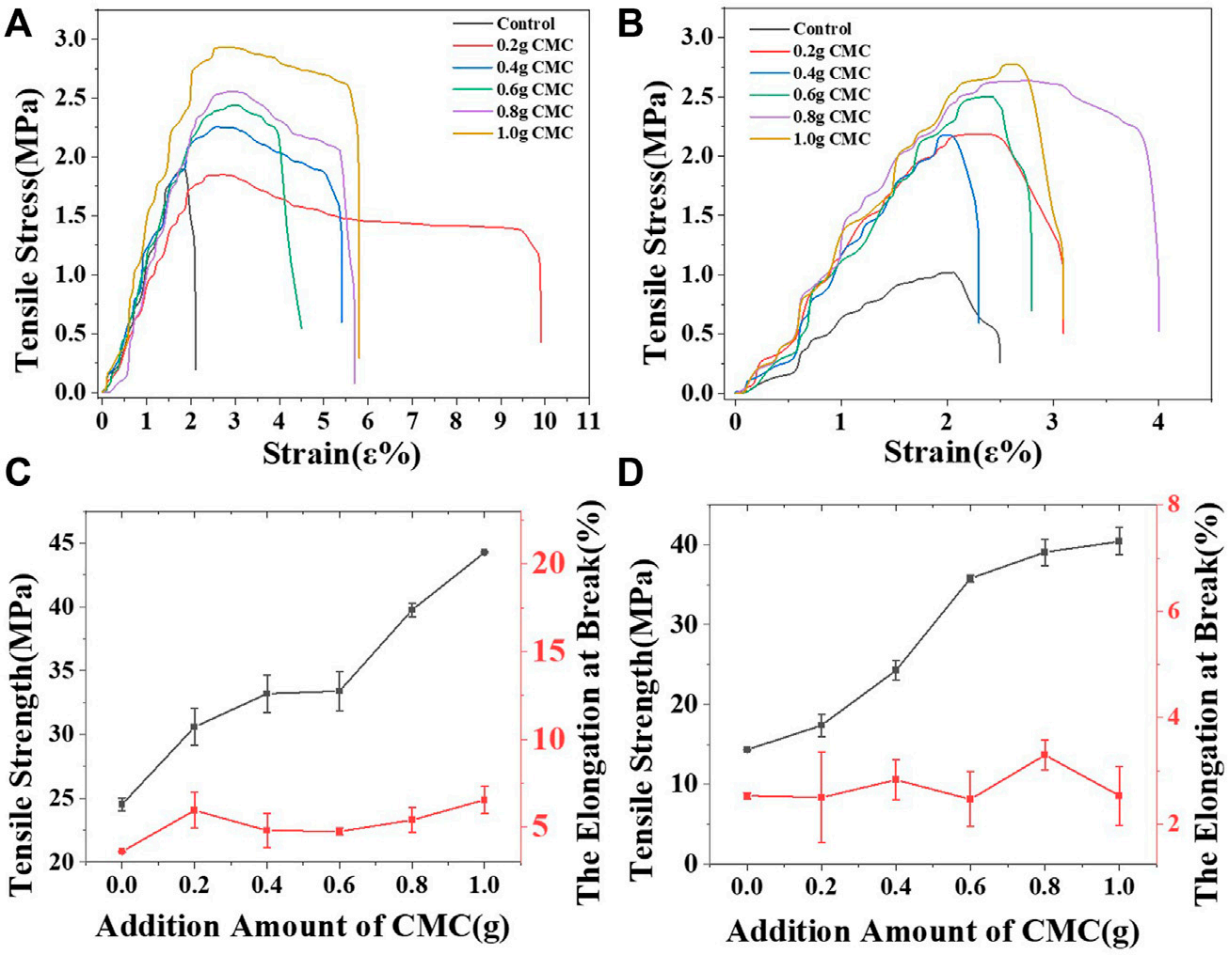
Stress-strain curve and the tensile strength with the elongation at break of the starch/CMC composite films. **(A, C)**: CS/CMC composite films; **(B, D)**: CA/CMC composite films.

Figures 2A, B shows the stress‒strain curve of the starch/CMC composite film; Figures 2C, D shows the tensile strength and elongation at break of the starch/CMC composite films. As shown in Figure 2, the tensile strength of the CS starch film was higher than that of the CA film, and the elongation at break of the CS films was greater than that of the CA films; this indicated that the mechanical properties of the CS film were better than those of the CA film. Both CMC composite films using 1.0 g of CMC had a large tensile strength of over 40 MPa. The elongation at break of the CS/CMC composite film was 6.55%, which was slightly higher than that of the pure CS film; this indicated that the film was correspondingly soft at this point. As more CMC was added, the tensile strength of both film materials increased. Similarly, the yield strength also showed a direct relationship with the amount of CMC added; as more CMC was added, the yield strength increased. The tensile strength of the CS film increased to 180.7% in comparison to the control group due to the addition of CMC, and the tensile strength of the CA film increased to 281.9%. The addition of CMC significantly increased the yield strength of the film and resulted in a considerable increase in the service life of the composite films. The analysis showed that new hydrogen bonds were formed between the -OH of starch and the -COO- of CMC; this enhanced the interaction between starch and CMC (Ou et al., 2021; Ma et al., 2020), improved the tensile strength of the starch/CMC composite films, and resulted in enhanced rigidity properties. In general, CMC improved the mechanical properties of starch films.

### 3.2 Morphological characterization

**FIGURE 3:**
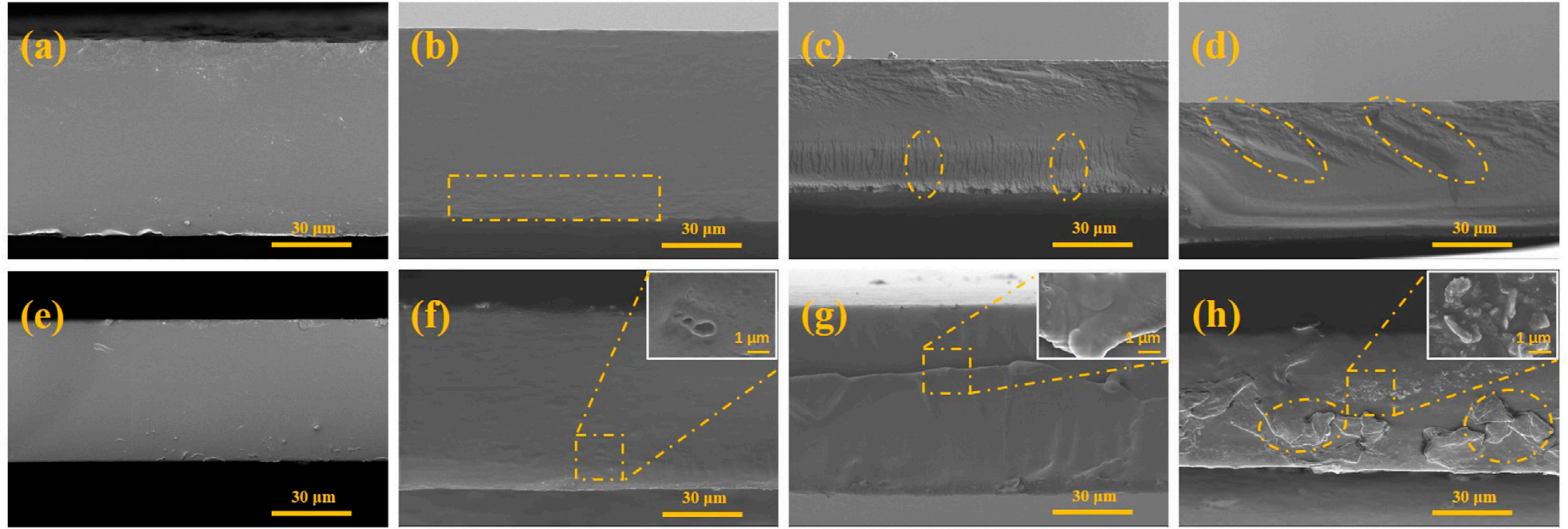
SEM of the starch/CMC composite films. **(A)**: pure CS film; **(B)**: CS/CMC-0.2 composite film; **(C)**: CS/CMC-0.6 composite film; **(D)**: CS/CMC-1.0 composite film; **(E)**: pure CA film; **(F)**: CA/CMC-0.2 composite film; **(G)**: CA/CMC-0.6 composite film; **(H)**: CA/CMC-1.0 composite film.

To understand the changes occurring inside the starch film, the cross-section was quenched at low temperature, and the morphology was observed (Figure 3). As shown in Figure 3, the smoothness of both unmodified starch films was good, while the cross-sectional roughness of the starch/CMC composite films increased as the amount of CMC added increased. Compared with the unmodified CS film, the ductile fracture traces of the CS/CMC composite film gradually became evident (Figures 3C,D). The CA/CMC composite films were rougher and more uneven than the pure CA film. With the addition of CMC, the cross-section of the CA/CMC composite film appeared uneven, and the incompatibility of the two phases was observed in Figure 3F; this was more apparent in the CA film with large amounts of CMC added, and caking caused by massive agglomeration appeared (Figure 3H). The analysis showed that CS was compatible with CMC, which enhanced the film. Figure 2 shows that CMC had a better enhancement effect on the CS film, while the enhancement effect on the CA film was not as good as that on the CS film. This was due to the agglomeration of the molecular chains produced by the large amount of CMC, which caused the cross-sectional quenched morphology of the CA/CMC-1.0 composite film to be uneven; lumpy agglomerates were observed The formation of new chemical bonds between CMC and amylopectin increased the crystalline zone; the distribution of the crystalline zone was heterogeneous, and the amylopectin and CMC were not homogeneous in both phases, thus explaining its weaker mechanical properties than the CS/CMC composite film.

### 3.3 XRD structure analysis

**FIGURE 4:**
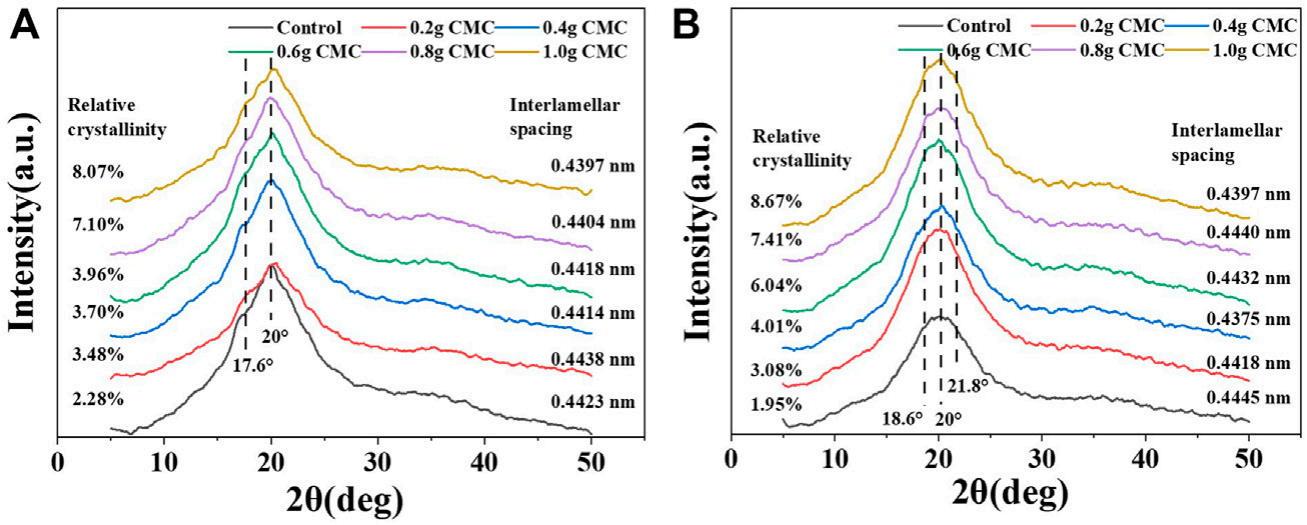
XRD curves of the starch/CMC composite films. **(A)**: CS/CMC composite films; **(B)**: CA/CMC composite films.

To understand the crystallinity of the starch film material, XRD analysis was performed (Figure 4). As shown in Figure 4, the starch film material showed crystalline peaks at 18°and 20°, and the CA film material showed crystalline peaks at 18.6°, 20.0° and 21.8°. Figure 4 shows that the addition of CMC changed the degree of crystallinity and layer spacing of the starch film. Based on the fitted crystallinity, the crystallinity was less than 10%, and the trend was that as CMC was added, the crystallinity of the starch film material increased; this indicated that the starch film material mainly exhibited amorphous characteristics and that the starch and CMC were largely converted to the amorphous state (Dolas et al., 2020). CMC reduced the layer spacing of the starch composite films.

The analysis showed that after the pasting and cooling process, the starch chains were recrystallized, and the molecular chains of starch were transformed from ordered to disordered to ordered structures. During cooling, the movement of the starch molecular chain was inhibited, and the system was in a thermodynamic non-equilibrium state. The restricted molecular chains rearranged due to hydrogen bonds, and then the starch recrystallized. The amylose in CS formed double helical enrichment regions, which eventually formed intermolecular hydrogen bonds. In contrast, recrystallization of amylopectin occurred between the branches, with more crystalline regions forming within the molecule. CMC is a chain structure and possesses sites for hydrogen bond formation with starch. At low dosages, the reaction sites between CMC and starch were limited and resulted in little difference in the crystalline regions; the CS and CA films were essentially the same. At a high dosage of CMC, the amylopectin, due to its branching structure, had more hydrogen bond formation sites that tightly attached to the CMC and caused its crystallinity to be greater than that of the CS film. The crystallinity of the two film materials were related the elevated mechanical properties of the starch films in Figure 2; these elevated properties were caused by the increased crystallinity.

### 3.4 Stability analysis

**FIGURE 5:**
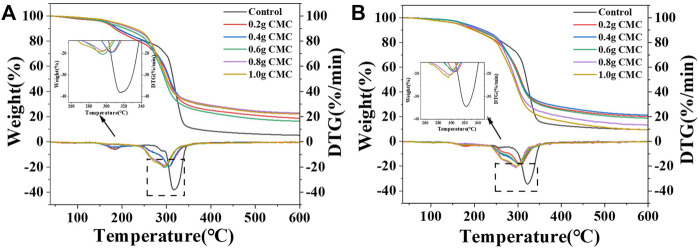
TG and DTG curves of the starch/CMC composite film. **(A)**: CS/CMC composite films; **(B)**: CA/CMC composite films.

The influence of CMC on the thermal stability of the two types of films were studied using TG analysis. The TG and DTG curves of the CS/CMC and CA/CMC composite films are shown in Figure 5. As shown in Figure 5; Table 1, with the addition of CMC, the temperature of the maximum weight loss rate of the two composite films decreased. The residual carbon rates of starch/CMC composite films were higher than those of the pure starch films. The analysis showed that the doping of CMC caused the composite films to degrade at a lower temperature because CMC introduced carboxyl groups on the starch surface and reduced the maximum weight loss rate temperature of the CS film (Meng et al., 2016). From the analysis of residual carbon rate data, the addition of CMC improved the thermal stability of the film; the addition of CMC increased the intermolecular bonding force of films and increased the number of hydrogen bonds formed between the two macromolecular chains of starch and CMC, which hindered the movement of the macromolecular chains and inhibited the pyrolysis of the films (Siqueira et al., 2010). This result was also verified by the increase in crystallinity. All residual carbon rates of the CS films increased due to the addition of CMC. The CA films started to decrease above the addition of .6 g probably because the crystallinity of the film material did not show a linear increase with the addition of CMC. Compared with a small amount of CMC, the excessive CMC did not significantly improve the crystallinity of the films. It was predicted that there was more CMC free in the amorphous zone of the film material, which caused the decrease in the residual carbon rate.

**TABLE 1:** Temperature of maximum weight loss rate (Tp) and residual carbon rate (RC) of the starch composite films.

Sample	Tp (°C)	RC (%)	Sample	Tp (°C)	RC (%)
CS	315.5	5.2	CA	322.9	9.5
CS/CMC-0.2 g	307.1	18.5	CA/CMC-0.2 g	306.2	20.1
CS/CMC-0.4 g	305.0	22.5	CA/CMC-0.4 g	304.1	21.3
CS/CMC-0.6 g	296.1	16.3	CA/CMC-0.6 g	301.1	18.8
CS/CMC-0.8 g	295.2	22.9	CA/CMC-0.8 g	298.2	13.3
CS/CMC-1.0 g	293.2	22.1	CA/CMC-1.0 g	295.2	9.5

**FIGURE 6:**
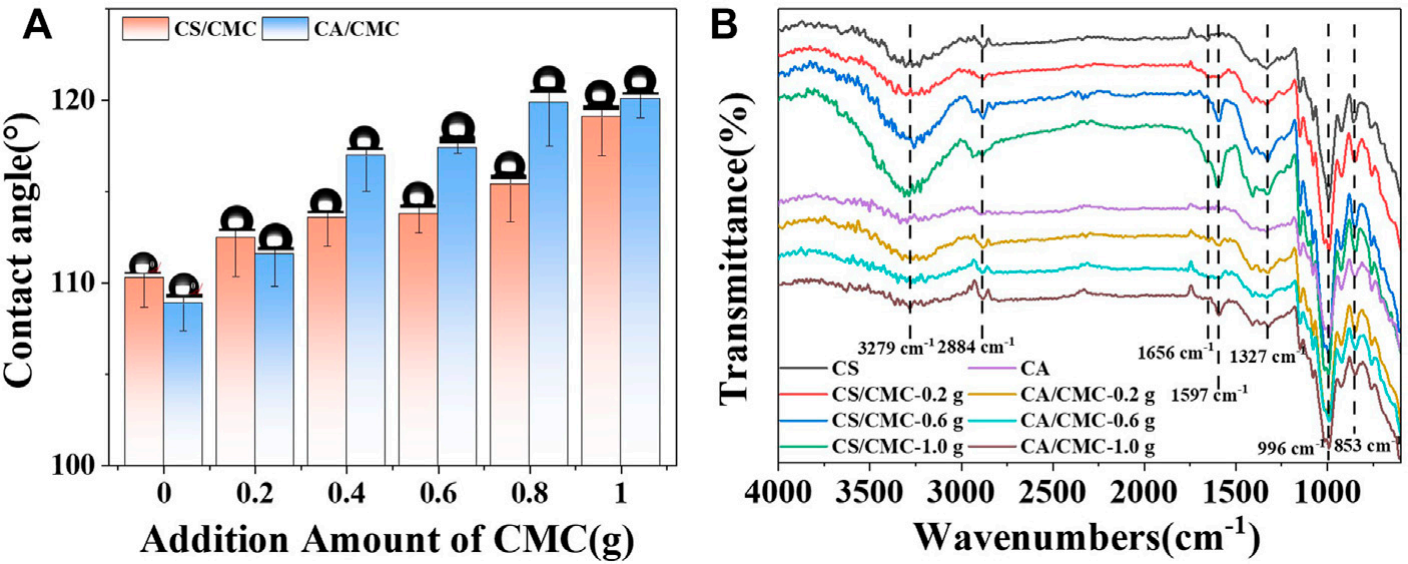
The contact angle and FTIR spectra of the starch/CMC composite films. **(A)**: the contact angle; **(B)**: FTIR spectra.

To investigate the effect of CMC on the hydrophobicity of the CS and CA composite film surfaces, water contact angle tests were conducted. As shown in Figure 6A, the water contact angle was 110.3° for the pure CS film material and 108.1° for the pure CA film. With the addition of CMC, the water contact angle of both starch films increased. When the addition amount reached 1.0 g, the water contact angle of the CS/CMC composite film was 119.1°; this was an 8% increase compared to the pure CS film and a 10.3% increase for the CA/CMC composite film. When the amount of CMC added was more than .4 g, the water contact angle of the CS/CMC composite film was greater than that of the CA/CMC composite film. The analysis showed that when CMC was added in small amounts, the hydrophilicity of their composite films was better because of the higher content of amylopectin in the films (Zhang et al., 2019). When more CMC was added, new hydrogen bonds were formed between CMC and CA, resulting in a tighter structure of the composite film and the destruction of the original structure; this exposed the hydrophobic groups to the surface of the film and caused the surface to be less hydrophilic (Jose et al., 2012). The reaction of starch molecules with CMC introduced carboxylic acid derivative ester groups, which increased hydrophobicity to some extent. This was consistent with the data on the crystallinity of starch.

### 3.5 Chemical structure analysis

To understand the effect of CMC on the starch film groups, FTIR analysis was performed (Figure 6B). The FTIR spectrum of the starch/CMC composite film showed a stretching vibration peak of -OH at 3,279 cm^−1^, a stretching vibration peak of -CH_2_ at 2,884 cm^−1^, and an absorption peak of C=O at 1,656 cm^−1^. The absorption peaks at 1,597 cm^−1^ and 1,327 cm^−1^ corresponded to carboxylate asymmetric stretching (Mansur et al., 2017), the absorption peak at 996 cm^−1^ corresponded to the stretching vibration absorption peak of C-O in C-O-C, and the absorption peak near 853 cm^−1^ corresponded to the C-C skeleton vibration (Zhang et al., 2013). In pure CS films, the peaks at 1,660–1725 cm^−1^ were attributed to the -OH bending vibration of water in the starch molecule (Ren et al., 2017; Shim et al., 2020). Analysis of Figure 6B shows that the two composite film materials exhibited new peaks at 1,656 cm^−1^ and 1,597 cm^−1^, indicating the formation of hydrogen bonds between CMC and starch and the introduction of carboxylic acid group derivatives (ester groups).

**FIGURE 7:**
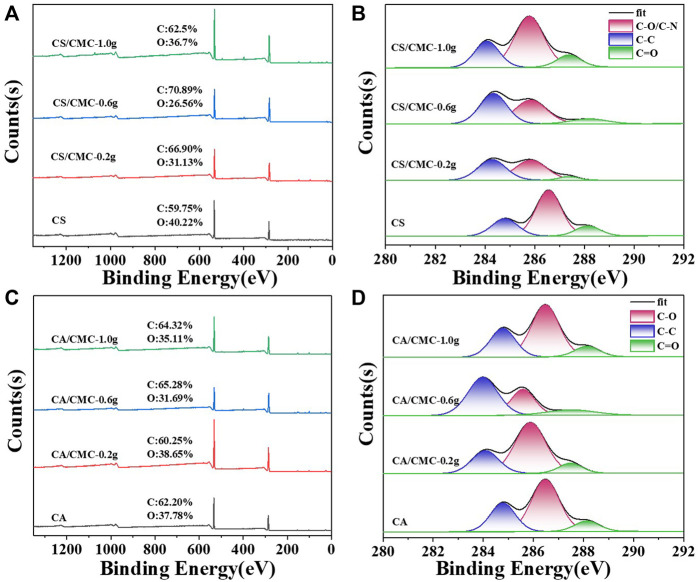
XPS survey spectra and high-resolution C1s spectra of the starch/CMC composite films. **(A, C)**: CS/CMC composite films; **(B, D)**: CA/CMC composite films.

X-ray photoelectron spectroscopy (XPS) analysis was conducted to obtain more in-depth information on the chemical bond composition and surface elements (Figure 7; Table 2). As shown in Figure 7, the total XPS spectra of the starch film samples all showed two distinct characteristic peaks: 286.6 eV (C1s) and 532.8 eV (O1s). The high-resolution XPS spectra of the C1s of the four film samples were further divided into three peaks. The first peak at 284.2 eV was attributed to C-C (sp^3^ carbon), while the other two peaks were located at binding energies of 286.0 eV and 287.6 eV and belonged to C-O and C=O in the starch and CMC chains, respectively (Yu and Kwak, 2012). Table 2 shows that the starch/CMC composite films showed an overall enhancement in the percentage of C elements compared to the pure starch films. When CMC was added, the proportion of C-C bonds in the composite films increased, while the proportion of C-O and C=O bonds decreased. In particular, the C=O bond in the CS/CMC-1.0 composite films accounted for only 11.66% of the C1s.

**TABLE 2:** Element proportion and chemical bond for the starch/CMC composite films.

Sample	C1s (%)	O1s (%)	C-C (%)	C-O (%)	C=C (%)
CS	59.75	40.22	24.84	62.11	13.04
CS/CMC-0.2	66.90	31.13	49.50	45.05	5.45
CS/CMC-0.6	70.89	26.56	43.19	46.95	9.86
CS/CMC-1.0	62.50	36.79	26.99	61.35	11.66
CA	62.20	37.78	34.08	55.87	10.06
CA/CMC-0.2	60.25	38.65	25.81	64.52	9.68
CA/CMC-0.6	65.28	31.69	56.81	30.68	12.50
CA/CMC-1.0	64.32	35.11	40.39	49.26	10.34

### 3.6 Electrochemical performance analysis

**FIGURE 8:**
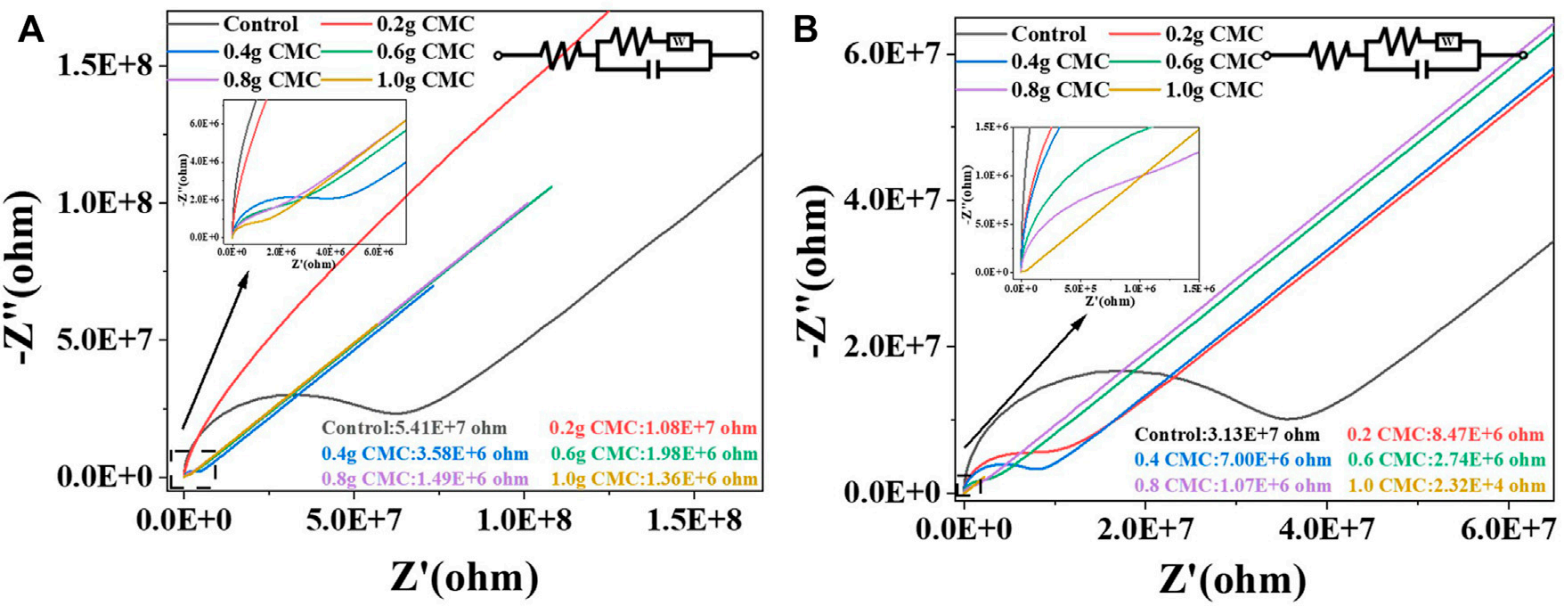
EIS of the starch/CMC composite films. **(A)**: CS/CMC composite films; **(B)**: CA/CMC composite films.

To assess the electrical conductivity of the films, the starch/CMC composite films were tested using electrochemical impedance spectroscopy (EIS). As shown in Figure 8, the EIS curve consists of a semicircle and a sloping straight line. The semicircular arc region in the plot is the charge transfer impedance due to gain and loss of electrons, while the straight line region is related to the solid-state diffusion process (Xu J. et al., 2018; Xu T. et al., 2018). The diameter of the semicircular arc region represents the magnitude of the resistance of the material. The circuit-fitted EIS profiles showed that the resistance values of the starch films all decreased as the CMC doping increased, and the resistances of the CA/CMC composite films varied more. The resistance of the pure CS film was 5.41E+7 Ω, while the resistance of the CS/CMC-1.0 composite film was only 1.36E+6 Ω. The resistance of the composite film decreased by one order of magnitude. When the doping amount of CMC was 1.0 g in the CA film, the resistance of the composite film decreased by two orders of magnitude compared to that of the pure CA film. This was due to the presence of Na^+^ in the CMC structure, which bound electrostatically to the oxygen-containing functional groups in the starch structure; this built a conductive pathway, provided a charge transfer pathway, and resulted in a better conductivity of the CMC-doped composite films (Cyriac et al., 2022).

To more clearly show the conductivity gap of the films, the current strength of the films in different pH solutions were tested. A sensitive pH response of the composite films was found in the variation of the currents. Different pH solutions caused a change in the current of the film, thus characterizing the acidity of the solution by the current.

**FIGURE 9:**
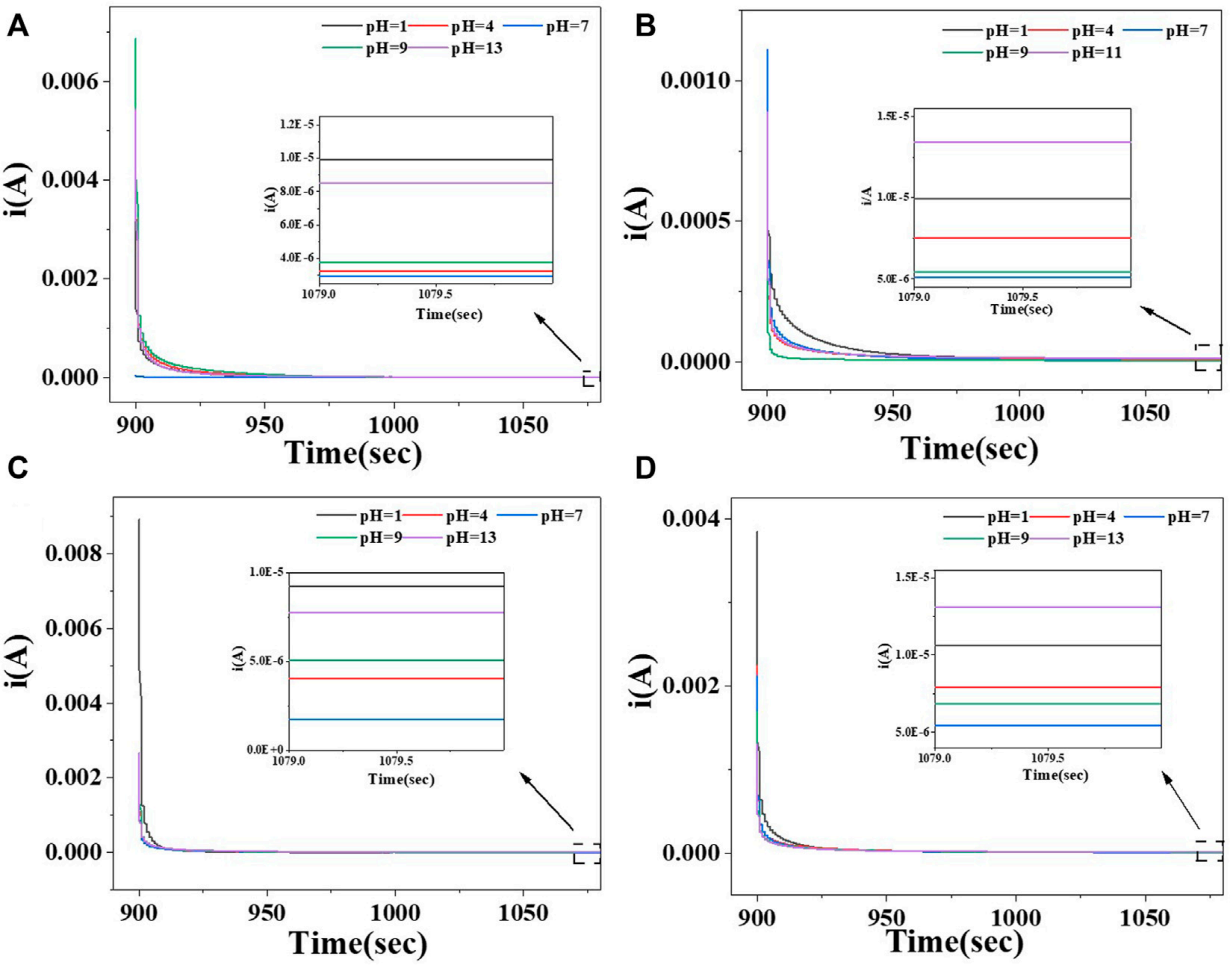
Steady current at different pH value of the starch/CMC-1.0 composite film. **(A)**: pure CS film; **(B)**: CS/CMC-1.0 composite film; **(C)**: pure CA film; **(D)**: CA/CMC-1.0 composite film.

As shown in Figure 9, the stable currents for pure CS films ranged from 2.88E-6 A to 9.76E-6 A at different pH values and 4.99E-6 A to 1.32E-5 A for CS/CMC-1.0 composite films; the stable currents for pure CA films ranged from 2.15E-6 A to 9.25E-6 A at different pH values and CA/CMC-1.0 composite films ranged from 5.27E-6 A to 1.29E-5 A. The four films showed the same trend of currents in different pH environments.

The analysis shown in Figure 9 revealed that the doping of CMC improved the electrical conductivity of the starch films; the current of the starch/CMC composite films was higher than that of the pure starch films in different environments due to the Na^+^ in the CMC structure. Under acidic or alkaline conditions, the number of ions that could be freed in the film was positively correlated with the current, and thus, the conductivity was better (Tao et al., 2021). According to the CA test results, the doping of CMC effectively reduced the resistance of the starch films and thus enhanced their electrical conductivity; this was consistent with the EIS result described above. The EIS and current of the starch films were comprehensively analyzed, and the current values of the CA films were greater than those of the CS films; this indicated that the CA films were more sensitive to variations in pH.

To verify the pH response of the starch/CMC composite films, we used orange juice after 14 days of storage as the test fluid. Orange juice was added dropwise to self-assembled devices prepared from starch/CMC composite films to test their stable current.

**FIGURE 10:**
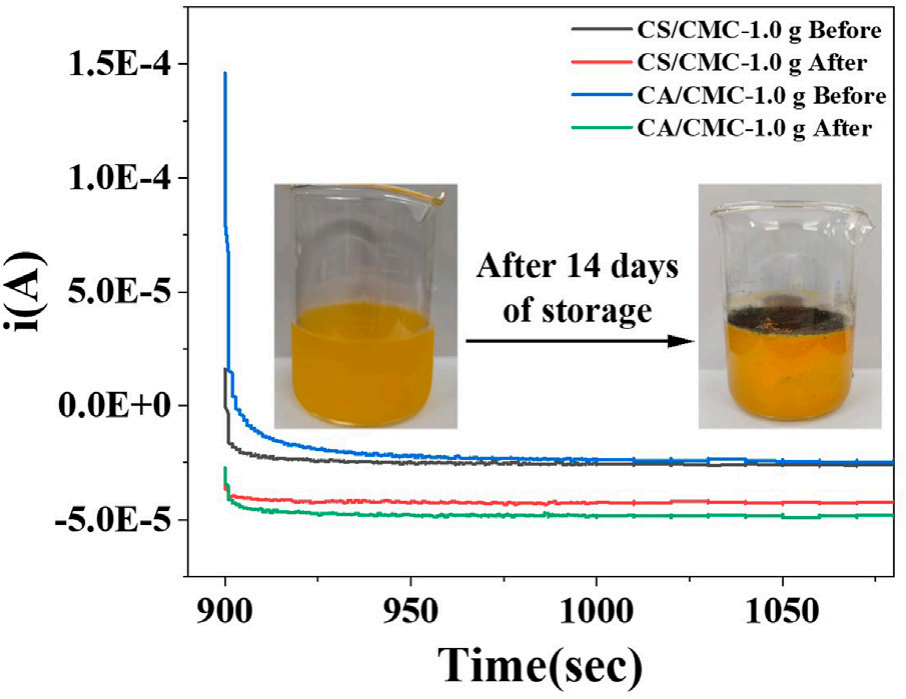
Steady current of the starch/CMC-1.0 composite films.

The initial pH of the fresh orange juice was 3.38, and the pH of the spoiled orange juice was 2.68. As microorganisms broke down the large molecules of acid in the orange juice into smaller ones, this increased the H^+^ in the orange juice, which in turn decreased the pH of the orange juice (Zhang et al., 2016). As shown in Figure 10, the stable currents measured by the CS/CMC-1.0 composite film before and after juice deterioration were −2.6E-5 A and −4.28E-5 A, respectively; the stable currents measured by the CA/CMC-1.0 composite film before and after juice deterioration were −2.55E-5 A and −4.83E-5 A, respectively. Comparing the test results of the two composite films, the starch/CMC composite films showed the same trend in current, and the difference in current before and after juice deterioration was similar. Using the current of the starch/CMC composite film device, it was found that the information on the pH change of orange juice before and after spoilage could be captured; the information on the spoilage of orange juice was obtained by combining the relationship between the pH value and the current (Figure 10). Through objective juice experiments, it was determined that the composite film had a current response of −2.88E-5 A order of magnitude, a sensitive pH response ability and application to the rapid detection of juice quality.

## 4 Conclusion

In conclusion, starch/CMC composite films with different CMC doping levels were prepared by solution casting methods, and their properties and pH responsiveness were evaluated. The tensile strength of the CS/CMC and CA/CMC composite films reached 44.27 MPa and 40.43 MPa, respectively. CMC doping increased the crystallinity of the starch films; the highest crystallinity was only 8.67%. Due to the high crystallinity of the recrystallized CA film, the stability of the CA films was better, and the temperature of the maximum weight loss rate was higher than that of the CS film. The doped CMC caused the starch films to be more hydrophobic, which laid a foundation for the reuse of liquid food testing materials. The starch/CMC-1.0 composite films had good electrical conductivity and sensitively responded to different pH solutions. The results showed that the current difference of the CA/CMC composite film was more evident, which was more conducive to the quality monitoring of juice. In general, the composite material prepared in this study is biodegradable since both starch and CMC are degradable. The acid-base responsive composite films in this work shows some promise for field applications of rapid food detection and biodegradable films and provides a new strategy for the quality detection of liquid food.

## Data Availability

The original contributions presented in the study are included in the article/Supplementary Material, further inquiries can be directed to the corresponding authors.
